# Supplementary material for: Phyllanthus niruri Linn.: Antibacterial Activity, Phytochemistry, and Enhanced Antibiotic Combinatorial Strategies
Source: Antibiotics (Basel). 2024 Jul 16;13(7):654. doi: 10.3390/antibiotics13070654 (PMC11273511; doi:10.3390/antibiotics13070654)
Supplement: Supplementary file 1 [file antibiotics-13-00654-s001.zip › antibiotics-3104388-supplementary.pdf]

Supplementary Data

Phyllanthus niruri Linn.: Antibacterial Activity, Phytochemistry and Enhanced Antibiotic Combinatorial Strategies

Gagan Tiwana [1], Ian E. Cock [2] and Matthew J. Cheesman [1,*]

[1] School of Pharmacy and Medical Sciences, Gold Coast Campus, Griffith University, Gold Coast 4222, Australia; g.tiwana@griffith.edu.au (G.T.); m.cheesman@griffith.edu.au (M.J.C.)

[2] School of Environment and Science, Nathan Campus, Griffith University, Brisbane 4111, Australia; i.cock@griffith.edu.au

* Correspondence: m.cheesman@griffith.edu.au; Tel.: +61-7-55529230

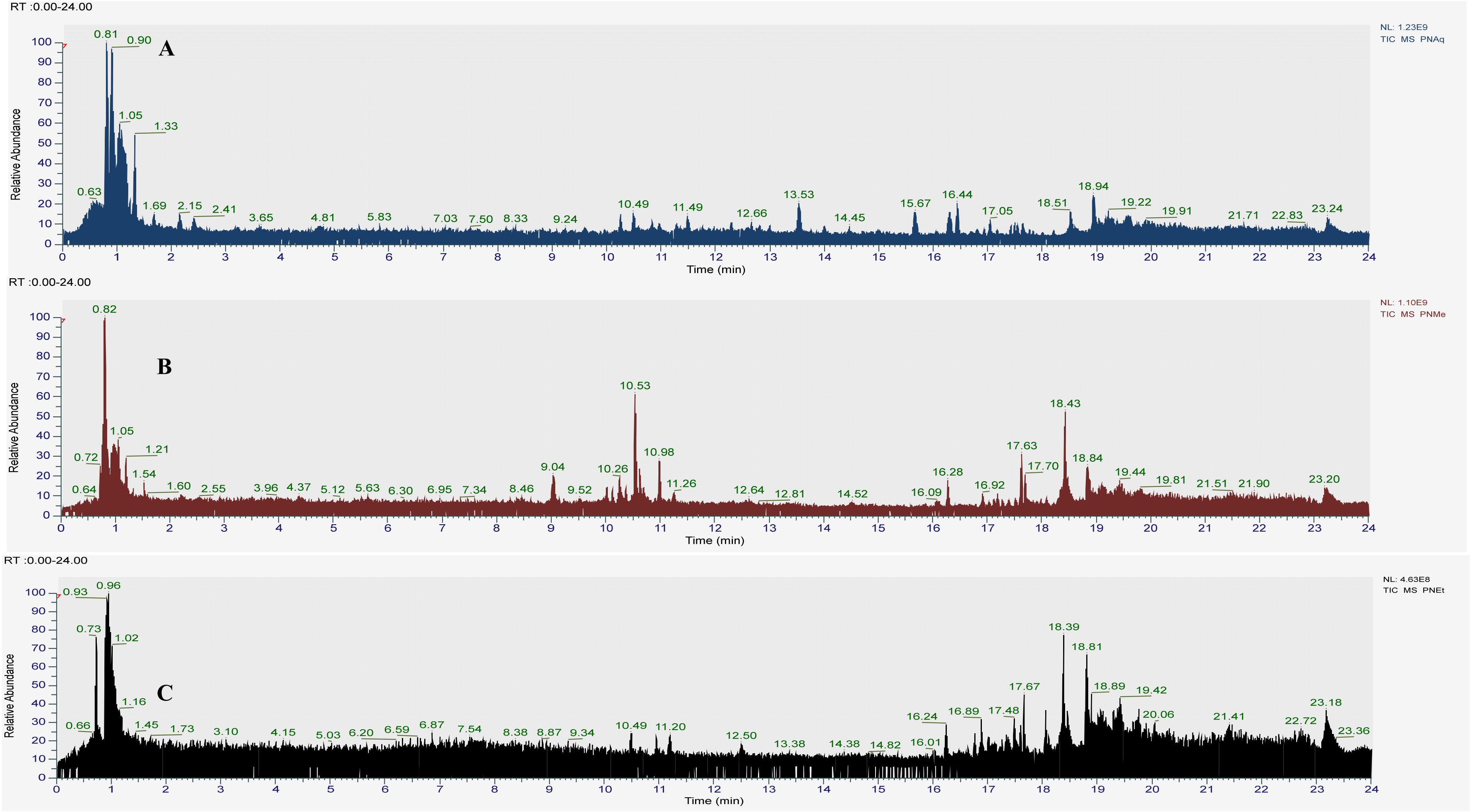

Supplementary Figure S1. LC-MS total compound chromatograms of (A) PN-Aq (*Phyllanthus niruri* aqueous), (B) PN-MeOH (*Phyllanthus niruri* methanol), and (C) PN-EtOAc (*Phyllanthus niruri* ethyl acetate) in negative ionisation mode.

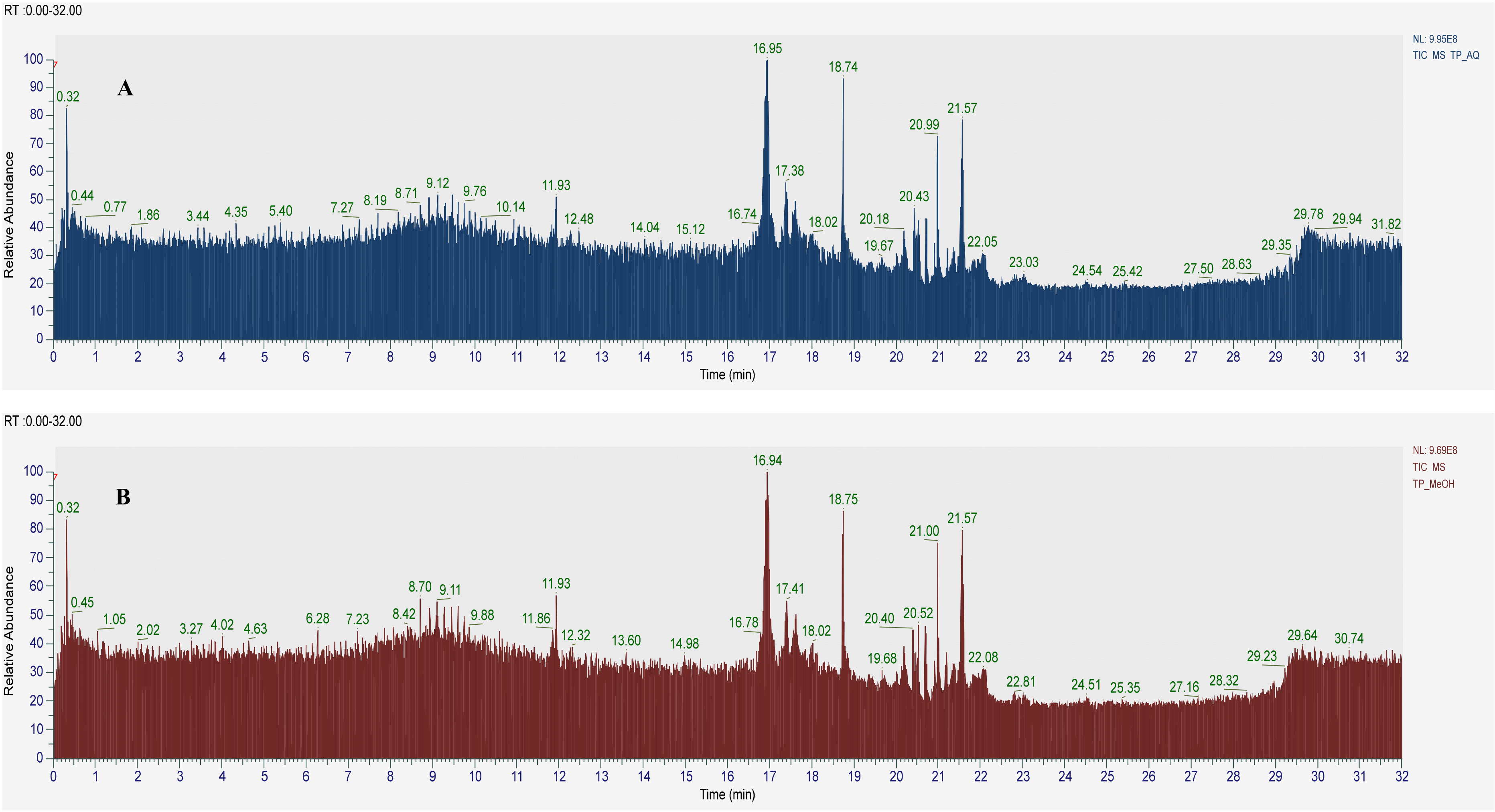

Supplementary Figure S2. LC-MS total compound chromatograms of (A) TP-Aq (*Terminalia phanerophlebia* aqueous), and (B) TP-MeOH (*Terminalia phanerophlebia* methanol) in negative ionisation mode.

Supplementary Table S1. LC-MS putative identification and % relative abundance of phytochemicals identified in the plant extracts.

Retention Time [min]	Molecular Mass	Empirical Formula	Putative Compounds	Relative Abundance (% of Total Area)		
				AQ	MeOH	EtOAc
**Isomers**						
1.416	192.02659	$C_6 H_8 O_7$	Isocitric acid	1.56%	1.07%	-
1.482	116.01078	$C_4 H_4 O_4$	Maleic acid	0.32%	0.43%	-
1.669	116.01081	$C_4 H_4 O_4$	Fumaric acid	-	0.10%	-
1.727	131.09453	$C_6 H_{13} N O_2$	Isoleucine	0.14%	0.27%	-
4.221	290.07887	$C_{15} H_{14} O_6$	Catechin	0.14%	0.27%	-
**Organic Compounds**						
1.383	103.0996	$C_5 H_{13} N O$	Choline	10.36%	6.57%	0.17%
1.389	182.07872	$C_6 H_{14} O_6$	L-Iditol	-	2.08%	-
1.4	342.11574	$C_{12} H_{22} O_{11}$	α, α-Trehalose	-	5.48%	-
1.404	196.05803	$C_6 H_{12} O_7$	Galactonic acid	-	0.83%	-
1.408	117.07863	$C_5 H_{11} N O_2$	Betaine	12.47%	11.79%	0.73%
1.411	205.03777	$C_{10} H_7 N O_4$	Xanthurenic acid	-	0.97%	-
1.436	104.01083	$C_3 H_4 O_4$	Malonic acid	2.88%	-	-
1.458	115.06324	$C_5 H_9 N O_2$	Proline	3.33%	-	-
1.46	135.05441	$C_5 H_5 N_5$	Adenine	-	0.31%	-
1.482	173.1051	$C_8 H_{15} N O_3$	N-Acetyl-L-leucine	0.05%	0.15%	-
1.513	112.01587	$C_5 H_4 O_3$	2-Furoic acid	-	0.23%	-
1.632	88.01578	$C_3 H_4 O_3$	Pyruvic acid	-	0.07%	-
1.652	181.07367	$C_9 H_{11} N O_3$	L-Tyrosine	-	0.25%	-
1.652	267.09639	$C_{10} H_{13} N_5 O_4$	Adenosine	-	1.17%	-
1.653	90.03155	$C_3 H_6 O_3$	L- (+)-Lactic acid	0.14%	0.11%	0.67%
1.657	145.07364	$C_6 H_{11} N O_3$	4-Acetamidobutanoic acid	0.10%	0.43%	-
1.657	129.04244	$C_5 H_7 N O_3$	L-Pyroglutamic acid	1.19%	1.63%	-
1.672	169.07365	$C_8 H_{11} N O_3$	Pyridoxine	0.31%	-	-
1.686	174.01631	$C_6 H_6 O_6$	trans-Aconitic acid	0.55%	-	-
1.779	148.03705	$C_5 H_8 O_5$	δ-Ribono-1,4-lactone	1.44%	-	-

1.78	118.02654	$C_4 H_6 O_4$	Succinic acid	2.21%	-	-
1.794	126.03153	$C_6 H_6 O_3$	Pyrogallol	5.39%	0.92%	-
1.799	122.03664	$C_7 H_6 O_2$	Benzoic acid	-	0.08%	-
1.805	131.09447	$C_6 H_{13} N O_2$	Leucine	-	0.29%	-
1.806	214.13145	$C_{10} H_{18} N_2 O_3$	Valylproline	0.17%	0.11%	-
2.06	166.0629	$C_9 H_{10} O_3$	Apocynin	-	0.04%	-
2.126	170.02141	$C_7 H_6 O_5$	Gallic acid	11.29%	0.18%	-
2.136	165.07879	$C_9 H_{11} N O_2$	L-Phenylalanine	-	0.10%	-
2.17	87.10474	$C_5 H_{13} N$	Isoamylamine	-	0.03%	-
2.206	154.02649	$C_7 H_6 O_4$	2,4-Dihydroxybenzoic acid	0.13%	0.05%	-
2.315	223.08432	$C_{11} H_{13} N O_4$	N-Acetyl-L-tyrosine	0.05%	-	-
2.429	99.06831	$C_5 H_9 N O$	N-Methyl-2-pyrrolidone	0.04%	-	-
2.518	219.11066	$C_9 H_{17} N O_5$	Pantothenic acid	0.19%	-	-
2.552	110.03666	$C_6 H_6 O_2$	Catechol	-	0.05%	-
2.638	152.04728	$C_8 H_8 O_3$	Resorcinol monoacetate	-	0.05%	-
2.688	154.02651	$C_7 H_6 O_4$	Protocatechuic acid	1.00%		-
2.803	192.02679	$C_6 H_8 O_7$	Citric acid	0.35%	0.55%	-
2.803	164.0473	$C_9 H_8 O_3$	4-Coumaric acid	0.30%	1.41%	-
2.847	244.0694	$C_9 H_{12} N_2 O_6$	Uridine	-		0.30%
3.162	187.06317	$C_{11} H_9 N O_2$	trans-3-Indoleacrylic acid	0.07%	0.76%	-
3.163	204.08964	$C_{11} H_{12} N_2 O_2$	DL-Tryptophan	-	0.08%	-
3.197	122.03665	$C_7 H_6 O_2$	4-Hydroxybenzaldehyde	0.69%	0.35%	-
3.212	228.14726	$C_{11} H_{20} N_2 O_3$	Prolylleucine	0.17%	0.18%	0.51%
3.386	164.05831	$C_8 H_8 N_2 O_2$	Ricinine	0.08%	0.09%	0.34%
3.525	210.03743	$C_6 H_{10} O_8$	D-Saccharic acid	2.29%	0.52%	-
3.799	138.03156	$C_7 H_6 O_3$	2,5-Dihydroxybenzaldehyde	0.06%	0.12%	-
3.87	228.14705	$C_{11} H_{20} N_2 O_3$	Leucylproline	-		0.46%
3.914	189.04244	$C_{10} H_7 N O_3$	Kynurenic acid	-	0.08%	-
4.087	484.08547	$C_{20} H_{20} O_{14}$	1,6-Bis-O-(3,4,5-trihydroxybenzoyl) hexopyranose	-	0.91%	-
4.161	154.02646	$C_7 H_6 O_4$	Gentisic acid	0.12%	-	-
6.000	136.05237	$C_8 H_8 O_2$	2-Methylbenzoic acid	0.12%		-

9.019	290.07884	$C_{15}H_{14}O_6$	Epicatechin	0.35%	0.59%	-
9.543	450.11616	$C_{21}H_{22}O_{11}$	3,5-Dihydroxy-2-(4-hydroxyphenyl)-4-oxo-3,4-dihydro-2H-chromen-7-yl hexopyranoside	0.12%	0.23%	-
9.545	288.06304	$C_{15}H_{12}O_6$	(-)-Fustin	-	0.13%	-
9.866	164.04724	$C_9H_8O_3$	2,3-Dihydro-1-benzofuran-2-carboxylic acid	-	0.24%	-
9.867	120.05741	C_8H_8O	Phenylacetaldehyde	0.77%	0.14%	-
9.882	302.00597	$C_{14}H_6O_8$	Ellagic acid	-	0.05%	-
10.575	164.04732	$C_9H_8O_3$	2-Hydroxycinnamic acid	0.59%	1.61%	-
10.639	448.1004	$C_{21}H_{20}O_{11}$	Orientin	-	0.12%	-
10.645	564.14774	$C_{26}H_{28}O_{14}$	Corymboside	-	0.06%	-
10.726	302.04228	$C_{15}H_{10}O_7$	2-(2,6-dihydroxyphenyl)-3,5,7-trihydroxy-4H-chromen-4-one	-	0.38%	-
10.726	610.15282	$C_{27}H_{30}O_{16}$	Quercetin 3-O-rhamnoside-7-O-glucoside	-	0.17%	-
10.744	271.17806	$C_{14}H_{25}NO_4$	4-Oxo-4-[(3-oxo-2-decanyl) amino] butanoic acid	-	-	0.94%
10.968	596.13747	$C_{26}H_{28}O_{16}$	2-(3,4-Dihydroxyphenyl)-5,7-dihydroxy-4-oxo-4H-chromen-3-yl 6-O-β-D-xylopyranosyl-β-D-glucopyranoside	0.04%	-	-
10.972	464.09493	$C_{21}H_{20}O_{12}$	Quercetin-3β-D-glucoside	-	0.08%	-
11.034	176.04724	$C_{10}H_8O_3$	4-Methylumbelliferone hydrate	0.44%	0.05%	-
11.036	174.08905	$C_8H_{14}O_4$	Suberic acid	-	0.06%	-
11.065	164.11997	$C_{11}H_{16}O$	Jasmone	0.04%	-	-
11.134	464.09502	$C_{21}H_{20}O_{12}$	Hyperoside	-	1.69%	0.70%
11.134	448.10015	$C_{21}H_{20}O_{11}$	2-(3,4-dihydroxyphenyl)-5,7-dihydroxy-3-{[(2S,3R,4R,5R,6S)-3,4,5-trihydroxy-6-methyloxan-2-yl] oxy}-4H-chromen-4-one	-	0.23%	-
11.217	432.1052	$C_{21}H_{20}O_{10}$	Vitexin	0.07%	0.32%	-
11.222	194.05787	$C_{10}H_{10}O_4$	Ferulic acid	0.20%	-	-
11.226	246.1001	$C_{13}H_{14}N_2O_3$	2-(acetylamino)-3-(1H-indol-3-yl) propanoic acid	0.06%	0.13%	-
11.227	290.08985	$C_{14}H_{14}N_2O_5$	Indole-3-acetyl-L-aspartic acid	0.05%	0.10%	-
11.231	478.0744	$C_{21}H_{18}O_{13}$	Miquelianin	1.46%	5.62%	-

11.264	204.11489	$C_{13}H_{16}O_2$	1-[4-hydroxy-3-(3-methylbut-2-en-1-yl) phenyl] ethan-1-one	0.70%	0.68%	-
11.299	464.09519	$C_{21}H_{20}O_{12}$	Myricitrin	-	0.69%	0.31%
11.339	386.1939	$C_{19}H_{30}O_8$	3-Hydroxy-3,5,5-trimethyl-4-(3-oxo-1-buten-1-ylidene) cyclohexyl β-D-glucopyranoside	-	0.15%	-
11.367	286.04738	$C_{15}H_{10}O_6$	Fisetin	-	0.20%	-
11.378	522.21017	$C_{26}H_{34}O_{11}$	Lariciresinol 4-O-glucoside	-	0.18%	-
11.5	610.15333	$C_{27}H_{30}O_{16}$	Rutin	-	12.00%	5.85%
11.588	448.10018	$C_{21}H_{20}O_{11}$	Trifolin	-	0.73%	0.98%
11.766	286.04747	$C_{15}H_{10}O_6$	Kaempferol	0.11%	2.43%	2.00%
11.783	448.10041	$C_{21}H_{20}O_{11}$	Astragalin	-	0.09%	-
11.873	226.1567	$C_{13}H_{22}O_3$	5-(6-hydroxy-6-methyloctyl)-2,5-dihydrofuran-2-one		0.13%	
12.021	138.03161	$C_7H_6O_3$	4-Hydroxybenzoic acid	1.21%	0.27%	-
12.155	208.07342	$C_{11}H_{12}O_4$	6-Hydroxy-8-methoxy-3-methyl-3,4-dihydro-1H-isochromen-1-one	-	0.04%	-
12.180	288.06322	$C_{15}H_{12}O_6$	2,4,6-Trihydroxy-2-(4-hydroxybenzyl)-1-benzofuran-3(2H)-one	0.08%	0.05%	-
12.198	152.12004	$C_{10}H_{16}O$	Citral	-	0.04%	-
12.198	332.18326	$C_{16}H_{28}O_7$	(2R,3R,4S,5S,6R)-2-{[(2E,6R)-6-hydroxy-2,6-dimethylocta-2,7-dien-1-yl] oxy}-6-(hydroxymethyl) oxane-3,4,5-triol	-	0.28%	-
12.371	188.10466	$C_9H_{16}O_4$	Azelaic acid	1.29%	0.80%	-
12.491	302.04225	$C_{15}H_{10}O_7$	2-(2,4-dihydroxyphenyl)-3,5,7-trihydroxy-4H-chromen-4-one	-	-	0.24%
12.579	244.13104	$C_{12}H_{20}O_5$	3,8,9-trihydroxy-10-propyl-3,4,5,8,9,10-hexahydro-2H-oxecin-2-one (herbarumin II)	0.35%	0.18%	0.65%
12.684	136.05231	$C_8H_8O_2$	4-Methoxybenzaldehyde	0.04%	-	-
12.705	338.09988	$C_{16}H_{18}O_8$	4-Methylumbelliferyl-α-D-glucopyranoside	0.14%	0.08%	-
12.746	138.10437	$C_9H_{14}O$	Isophorone	0.14%	-	-
12.866	192.0785	$C_{11}H_{12}O_3$	4-oxo-5-phenylpentanoic acid	0.35%	0.17%	-
13.219	302.04249	$C_{15}H_{10}O_7$	Quercetin	0.42%	7.68%	2.37%
13.222	264.13583	$C_{15}H_{20}O_4$	Ambrosic acid	-	0.05%	0.44%

13.314	462.24631	$C_{22}H_{38}O_{10}$	2-(4-Methyl-3-cyclohexen-1-yl)-2-propanyl 6-O-(6-deoxy-α-L-mannopyranosyl)-β-D-glucopyranoside	-	0.04%	-
13.431	202.12037	$C_{10}H_{18}O_4$	3-tert-Butyladipic acid	-	0.12%	0.39%
14.083	304.16733	$C_{18}H_{24}O_4$	3,12-Dihydroxy-13-methoxypodocarpa-8,11,13-trien-7-one	-	0.09%	-
14.194	270.05268	$C_{15}H_{10}O_5$	Apigenin	-	0.05%	-
15.112	194.13053	$C_{12}H_{18}O_2$	Sedanolide	0.05%	0.10%	-
17.007	252.17238	$C_{15}H_{24}O_3$	Ageratriol	0.26%	-	0.37%
17.135	328.2249	$C_{18}H_{32}O_5$	Corchorifatty acid F	3.65%	2.69%	11.24%
17.185	330.24055	$C_{18}H_{34}O_5$	(15Z)-9,12,13-Trihydroxy-15-octadecenoic acid	-	0.75%	2.09%
17.291	294.2193	$C_{18}H_{30}O_3$	13(S)-HOTrE	0.19%	-	
17.331	210.12544	$C_{12}H_{18}O_3$	Jasmonic acid	0.51%	-	0.26%
17.492	298.15663	$C_{19}H_{22}O_3$	Ostruthin		0.02%	
17.558	216.1512	$C_{15}H_{20}O$	(+)-ar-Turmerone	0.03%	-	0.23%
17.685	268.07334	$C_{16}H_{12}O_4$	7-hydroxy-3-(4-methoxyphenyl)-4H-chromen-4-one	-	-	0.58%
17.694	294.1826	$C_{17}H_{26}O_4$	(+/-)-Gingerol	0.20%		
17.969	312.22993	$C_{18}H_{32}O_4$	(±)9-HpODE	1.44%	0.27%	1.37%
18.111	292.20361	$C_{18}H_{28}O_3$	12-oxo Phytodienoic Acid	0.08%	0.31%	2.57%
18.225	162.06795	$C_{10}H_{10}O_2$	4-Methoxycinnamaldehyde	0.07%	0.56%	0.85%
18.226	134.07305	$C_9H_{10}O$	2,4-Dimethylbenzaldehyde	-	0.22%	0.94%
18.344	228.07843	$C_{14}H_{12}O_3$	8,8-dimethyl-2H,8H-pyrano[3,2-g] chromen-2-one	-	0.05%	-
18.35	278.22405	$C_{18}H_{30}O_2$	α-Eleostearic acid	0.05%	-	-
18.462	286.08382	$C_{16}H_{14}O_5$	(2E)-3-(3,4-dihydroxy-2-methoxyphenyl)-1-(4-hydroxyphenyl) prop-2-en-1-one (Licochalcone B)	-	-	0.17%
18.494	388.20944	$C_{19}H_{32}O_8$	4-(4-hydroxy-2,6,6-trimethyl-3-{[(2R,3R,4S,5S,6R)-3,4,5-trihydroxy-6-(hydroxymethyl) oxan-2-yl] oxy} cyclohex-1-en-1-yl) butan-2-one	-	0.05%	-
19.05	322.17789	$C_{18}H_{26}O_5$	α-Zearalanol	-	-	2.56%
19.5	414.20407	$C_{24}H_{30}O_6$	Bis(4-ethylbenzylidene) sorbitol	-	-	9.54%
